# Carbon Dots Derived from Tea Polyphenols as Photosensitizers for Photodynamic Therapy

**DOI:** 10.3390/molecules27238627

**Published:** 2022-12-06

**Authors:** Yuxiang Yang, Haizhen Ding, Zijian Li, Antonio Claudio Tedesco, Hong Bi

**Affiliations:** 1 School of Chemistry and Chemical Engineering, Anhui University, 111 Jiulong Road, Hefei 230601, China; 2 School of Materials Science and Engineering, Anhui University, 111 Jiulong Road, Hefei 230601, China; 3 Department of Chemistry, Center of Nanotechnology and Tissue Engineering−Photobiology and Photomedicine Research Group, Faculty of Philosophy, Sciences and Letters of Ribeirão Preto, University of São Paulo, São Paulo 14040-901, Brazil

**Keywords:** carbon dots, tea polyphenols, photosensitizer, photodynamic, biocompatibility

## Abstract

Photodynamic therapy (PDT) has become an emerging cancer treatment method. Choosing the photosensitizer (PS) compounds is one of the essential factors that can influence the PDT effect and action. Carbon dots (CDs) have shown great potential as photosensitizers in PDT of cancers due to their excellent biocompatibility and high generation of reactive oxygen species (ROS). Here, we used tea polyphenol as raw material for synthesized tea polyphenol carbon dots (T−CDs) that show dual emission bands of red and blue fluorescence and can efficiently generate hydroxyl radicals (OH) under mildly visible irradiation with a LED light (400–500 nm, 15 mW cm^−2^). The extremely low cytotoxicity and excellent biocompatibility of T−CDs without light irradiation were tested using MTT and hemolytic assay. Further, T−CDs have been shown by in vivo experiments, using a mouse breast cancer cell line (4T1) subcutaneously injected in the back of the mouse buttock as a model, to effectively inhibit the tumor cell proliferation in solid tumors and show an excellent PDT effect. In addition, pathological sections of the mice tissues after further treatment showed that the T−CDs had no apparent impact on the major organs of the mice and did not produce any side effect lesions. This work demonstrates that the as−synthesized T−CDs has the potential to be used as a PS in cancer treatment.

## 1. Introduction

Photodynamic therapy (PDT), another new light−stimulated cancer treatment method, has the advantages of fewer side effects, less damage to normal tissues and less drug resistance compared with traditional treatment methods (such as surgical resection, chemotherapy, and radiotherapy) and could work coadjuvantlye with other therapies [1,2,3,4,5,6]. It is a promising treatment protocol. In PDT, a photosensitizer (PS) compound is one of the critical elements for the therapeutic effect. After being stimulated by visible light, it will photosensitively react with the surrounding biological environment to generate reactive oxygen species (ROS), thereby increasing target cell cytotoxicity and causing tissue damage and even death. There are two types of photoreactions to produce ROS. The type I mechanism is about electron transfer reaction, which generates hydroxyl radicals (·OH), hydrogen peroxides (H_2_O_2_), and superoxide anions (·O_2_^−^). The type II mechanism is about energy transfer reaction, which produces singlet oxygen (^1^O_2_) [1,7]. Most of the existing first− and second− generation PSs are porphyrins, phthalocyanines, and their derivatives compounds, which are often limited by factors such as poor resistance to photobleaching under light irradiation, heavy metals effect, and low clearance rate from the target tissue [8]. Carbon dots (CDs) have been presented as a new metal−free carbon nanomaterial due to their abundant surface groups [9,10], superb biocompatibility, good photobleaching resistance, and adjustable optical properties [11], and many researchers’ works have conducted a series of exploration and attempts at its application in the field of PDT for different types of cancer. Excitingly, it was Ge et al. [12] who discovered for the first time that the synthesized graphene quantum dots (GQDs) have high ^1^O_2_ yield under light stimulation and are further applied to PDT tumor therapy in living mice. Jia et al. [13] prepared CDs−based nanospheres of about 50 nm by ion assembly using positively charged individual CDs as the basis and negatively charged sodium dodecyl benzene sulfonate (SDBS) as the cross−linker. Using the assembled nanospheres as a PDT photosensitizer for NIR response, the nanospheres have better near−infrared (NIR) absorption and ROS yield.

However, so far, only a few CDs have been reported in the literature to exhibit intrinsic ROS−generating capacity [14,15]. Therefore, many PDT studies on CDs use them as carriers −to load classical PS molecules and then apply CDs−based composites to PDT cancer therapy. For example, Lin et al. [16] combined the photosensitizer chlorin e6 (Ce6) with amino−functionalized red CDs to synthesize Ce6−modified red CDs (Ce6−RCDs). Besides excellent photostability and biocompatibility, its cancer therapeutic effect is significantly improved compared with pure Ce6 and CDs. Additionally, doping metal elements into CDs is an effective method to realize their ROS−generating capacity. For example, Jia et al. [17] successfully synthesized an Mn−doped Mn−phthalocyanine manganese phthalocyanine as a precursor. Mixed CDs (Mn−CDs). Mn−CDs have both bright NIR fluorescence and good T1−weighted magnetic resonance signals. More interestingly, Mn−CDs can not only efficiently generate ROS but also truly catalyze the generation of oxygen from hydrogen peroxide (H_2_O_2_) to overcome the influence of the hypoxic tumor microenvironment on PDT therapy, and achieve high multimodal imaging and efficient in vitro/in vivo tumor PDT therapy. Instead of using CDs as a new generation of photosensitizers to take advantage of their advantages, post−modified photosensitizers frequently use them as carriers. Hence, developing CDs that absorb light in the red or near−infrared region without any post−processing and have high ROS generation capacity is worthy of further study.

Tea polyphenols are a general term for polyphenols extracted from tea, and their anti−mutation effect in vitro can effectively reduce the occurrence of cancer [18,19]. Moreover, epigallocatechin gallate (EGCG), the main component of tea polyphenols, has been listed as a potential new anticancer drug because of its ability to specifically inhibit the growth of tumor cells, and has been paid attention to and studied by countries around the world [20]. It was previously reported that green tea was used as raw material to prepare CDs with high water solubility, good stability, high biocompatibility, and biosafety. Besides, the prepared CDs could effectively inhibit breast cancer cells [21].

Herein, inspired by the multiple excellent properties of tea polyphenols against cancer, water−soluble T−CDs with red emission were prepared by the solvothermal synthesis in formamide using citric acid and tea polyphenols as starting materials. Under different excitation wavelengths in the range of 360–620 nm, the T−CDs showed a broad absorption in the visible region and exhibited dual emission of blue and red fluorescence. More interestingly, we found that the T−CDs can efficiently generate hydroxyl radicals under the excitation of LED light (400–500 nm, 15 mW cm^−2^). In vitro/in vivo experiments proved that it has a good PDT therapeutic effect under the LED irradiation. Furthermore, due to the metal−free property of T−CDs, the hemolytic test, cell activity test and histopathological section demonstrated that the T−CDs have excellent biocompatibility and extremely low cytotoxicity. Therefore, T−CDs reported in this paper has the potential to be used as a PS for performing type I PDT.

## 2. Results and Discussion

### 2.1. Characterization of the T−CDs

**Figure 1:**
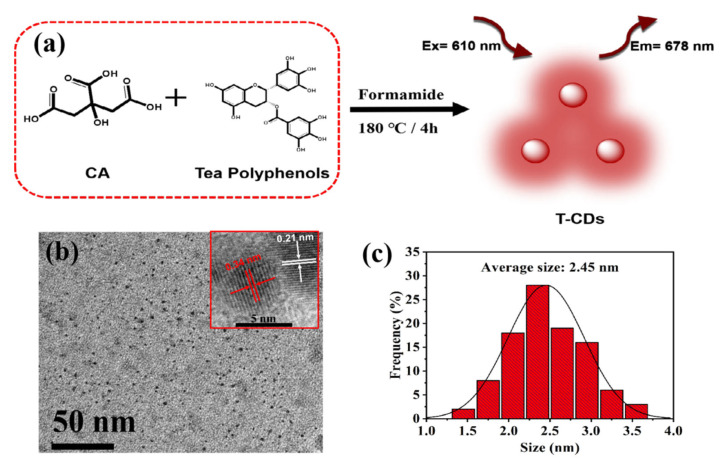
(**a**) Schematic diagram of the synthesis procedures and optical properties of T−CDs; (**b**) a typical TEM image (inset: HRTEM image); and (**c**) the corresponding particle size distribution histogram of T−CDs.

As shown in Figure 1a, the T−CDs were synthesized by citric acid (CA) and tea polyphenols in a formamide solvent at 180 °C for 4 h. To study the morphology and structure of T−CDs, we characterized the T−CDs by TEM. As shown in Figure 1b, the T−CDs have a spherical−like structure, and the dispersion is relatively uniform without aggregation. According to the corresponding particle size distribution histogram analysis (Figure 1c), the size distribution range of T−CDs was 1.3–3.7 nm, and the average particle size was 2.45 nm. TEM demonstrated that T−CDs were successfully prepared. The inset of Figure 1b shows the HR−TEM image of a single T−CDs, which shows that the carbon core of T−CDs has noticeable lattice fringes with two lattice spacings of 0.21 nm and 0.34 nm, corresponding to graphite. The in−plane (100) crystal plane and the graphite interlayer distance [22,23].

**Figure 2:**
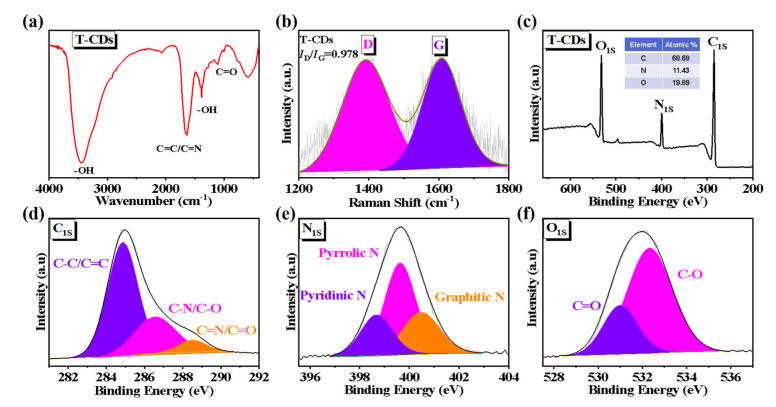
(**a**) FT−IR, (**b**) Raman spectra and (**c**) XPS survey of the T−CDs. XPS high−resolution (**d**) C 1s, (**e**) N 1s and (**f**) O 1s spectra of the T−CDs.

To further analyze the surface structure and elemental composition of T−CDs, we analyzed them by FT−IR, Raman spectroscopy, and XPS, respectively. An FT−IR spectrum for T−CDs is shown in Figure 2a, indicating that the absorption peak at 3432 cm^−1^ is related to the −OH and −NH on the surface of T−CDs (the amide bond in the reaction solvent formamide is involved in the reaction) [24]; at 1635 cm^−1^, the stretching vibration of C=C and C=N bonds is attributed to the absorption peak [25]; 1376 cm^−1^ may be attributed to the residual −OH of the polyphenol group in tea polyphenols [26], while 1107 cm^−1^ may be attributed to the stretching vibration of C=O. In addition, the Raman spectra of T−CDs (Figure 2b) exhibit two classical characteristic absorption bands, corresponding to the G−band peak of sp^2^ hybrid carbon (1603 cm^−1^) and the D−band peak of sp^3^ hybrid carbon (1603 cm^−1^). Among them, the intensity ratio of D−band to G−band (I_D_/I_G_) is a standard method used to evaluate the degree of graphitization and amorphous, disordered carbon structure in carbon materials [27]. Here, the I_D_/I_G_ of T−CDs is determined to be 0.978, which demonstrates the existence of abundant oxygen− and nitrogen−containing functional groups on the surface of T−CDs, which is consistent with the results of FT−IR. In addition, we determined the molecular weight (Mw) of T−CDs to be 60,739 daltons (Da) by gel permeation chromatography (GPC) (Figure S1). It is proved that T−CDs with a high molecular weight are formed by the dehydration, carbonization, and cross−linking polymerization of tea polyphenols and small citric acid molecules under high temperature and high−pressure reaction conditions.

To analyze the element content in T−CDs and the chemical environment of each element in more detail, we performed XPS spectroscopy on T−CDs. The full XPS spectrum, as shown in Figure 2c, reveals the atomic proportion of each element analyzed from the XPS data, in which C is 68.68%; N is 11.43%, and O is 19.89%, which indicates that the surface of T−CDs is rich in oxygen and nitrogen−containing functional groups. The chemical states of C, N, and O were analyzed in detail by the high−resolution XPS spectra of each element. As shown in Figure 2d, the C 1s band can be fitted to three constituent peaks corresponding to C−C/C=C (284.9 eV), C−N/C−O (286.6 eV) and C=N/C=O (288.6 eV) [28]. The high−resolution spectrum of N 1s (Figure 2e) also revealed three component peaks at 398.6 eV, 399.6 eV, and 400.5 eV, corresponding to pyridine N, pyrrolic N, and graphitic N in the carbon core, [29]. Figure 2f is the O 1s high−resolution spectrum of T−CDs, indicating two component peaks of C=O (531.0 eV) and C−O (532.3 eV) [30]. XPS data showed that T−CDs contained more O than C−O, indicating that polyphenolic groups of tea polyphenols were on their surfaces, which was attributed to their higher O content.

### 2.2. Optical Properties of the T−CDs

**Figure 3:**
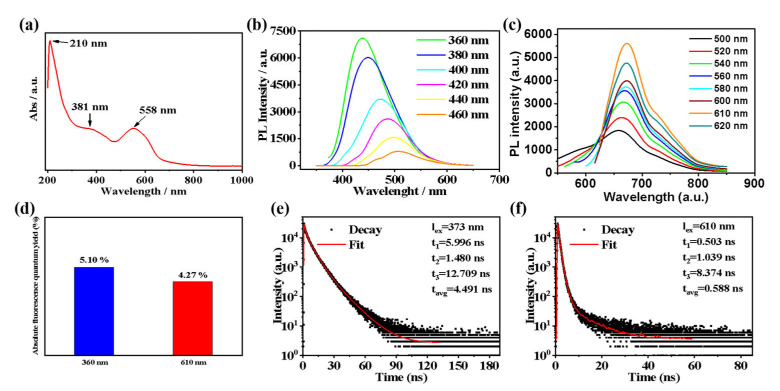
(**a**) UV−vis absorption and PL spectra under (**b**) 360–460 nm and (**c**) 500–620 nm excitation wavelengths of T−CDs in water. (**d**) Absolute fluorescence quantum yield of T−CD s in H_2_O under 360 nm and 610 nm excitations; Lifetime decay profiles of T−CDs in H_2_O under (**e**) 360 nm and (**f**) 610 nm excitations.

As shown in Figure 3a, T−CDs exhibit broad absorption in the UV−visible region, and their absorption sidebands extend into the near−infrared region. The UV absorption spectrum shows three main absorption peaks, and the two absorption peaks at 210 nm and 381 nm can be attributed to the π→π * and n→π * transitions of the sp^2^ hybrid structure in T−CDs, respectively [31]. Meanwhile, the broad absorption peak with the absorption center at 558 nm in the visible region may originate from the introduction of amide bonds in the solvent during the formation of T−CDs, which increases the conjugation plane and further expands the absorption range [32]. Figure 3b,c is T−CDs’ fluorescence emission spectra at different excitation wavelengths. It is noteworthy that T−CDs exhibited blue and red dual−color fluorescence emission behaviors when excited at two wavelengths ranging from 360–460 nm and 500–620 nm, respectively. It shows an excitation−dependent blue emission when the excitation wavelength is increased from 360 nm to 460 nm, and the optimal excitation wavelength is 360 nm, with the strongest emission at 436 nm. Besides, under the excitation wavelength of 500–620 nm, T−CDs exhibited a red emission with no apparent excitation dependence (the optimal excitation wavelength was 610 nm, and the maximum emission peak was located at 673 nm).

Under optimal excitation light, the QY of blue and red emissions of T−CDs is 5.10% (λ_ex_ = 360 nm) and 4.27% (λex = 610 nm), respectively (Figure 3d and Figure S2). The average fluorescence lifetime of the blue emission of T−CDs is 4.491 ns, according to the fluorescence lifetime decay curve (Figure 3e). Due to the increase in emission wavelength, the fluorescence emission lifetime in the red area is much shorter, with an average lifespan of 0.588 ns (Figure 3f). Thus, T−CDs have the potential for bioimaging in the red region due to their excellent fluorescence emission in aqueous solution.

### 2.3. ROS Production of the T−CDs

**Figure 4:**
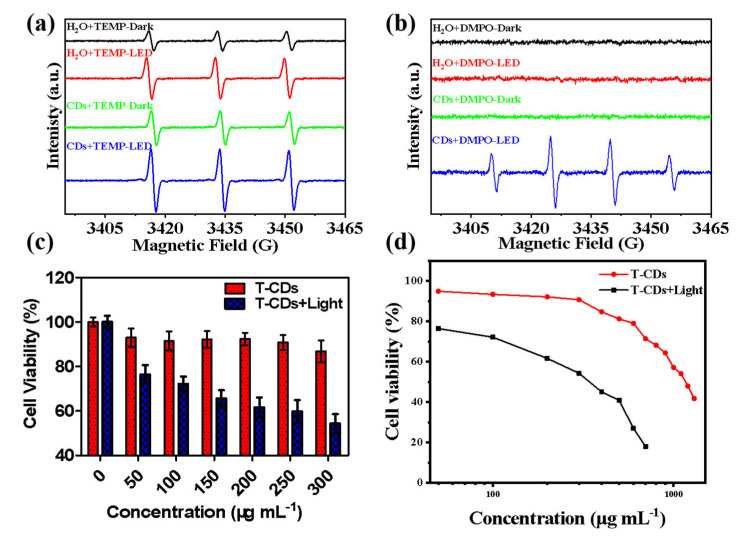
EPR spectra of H_2_O and T−CDs under LED irradiation, (**a**) TEMP as ^1^O_2_ trapper and (**b**) DMPO as ·OH trapper. Samples are irradiated by LED (400–500 nm, 15 mW cm^−2^) for 12 min. (**c**) MTT results of the 4T1 cells treated with T−CDs in the dark and under LED light irradiation (12 min), (**d**) The half maximal inhibitory concentration (IC50) values for T−CDs with or without light against 4T1 cell lines by MTT assay.

Electron paramagnetic resonance spectroscopy (EPR) is a standard method to analyze the ability and species of reactive oxygen species produced by light−induced photosensitive materials [33,34]. The EPR data revealed that both T−CDs and H_2_O showed typical ^1^O_2_ peaks, after 12 min of LED irradiation, the signal intensity of T−CDs was only weakly enhanced, indicating that the ability of T−CDs to generate ^1^O_2_ was weak under light (Figure 4a). However, using DMPO as the scavenger for ·OH, we found that only T−CDs showed a clear OH signal peak after being illuminated by LED, and the signal was significantly enhanced (Figure 4b). It can be seen that T−CDs have an excellent ability for light−induced ·OH generation. Hence, we speculate that T−CDs could be used for PDT, and in vivo/in vitro photodynamic therapy effects can be further studied. Then, we selected mouse cancer cell (4T1) as a model to evaluate the in vitro PDT effect of T−CDs. Meanwhile, the cytotoxicity of different concentrations of T−CDs under dark and LED light conditions has been detected by MTT assay (Figure 4c and Figure S3). We observed that T−CDs were not significantly toxic to 4T1 cells under dark conditions, and even at a high concentration of 300 μg mL^−1^, cell viability was still above 90%. Compared with the light group, it can be found that the cytotoxicity increased significantly after 12 min of LED light, and the cell viability decreased by 20% at the low concentration of 50 μg mL^−1^. Only around 50% of the cells were still viable after irradiation when the T−CDs concentration was 300 μg mL^−1^. Additionally, Figure 4d displays the half maximum inhibitory concentrations (IC50) for T−CDs against the 4T1 cell line as determined by the MTT experiment. The IC50 values of T−CDs with or without light were 344 µg mL^−1^ and 1155 µg mL^−1^, respectively. These results reveal that T−CDs exhibit the ability to generate OH and apparent cytotoxicity under LED irradiation.

### 2.4. Biosafety Testing of the T−CDs

**Figure 5:**
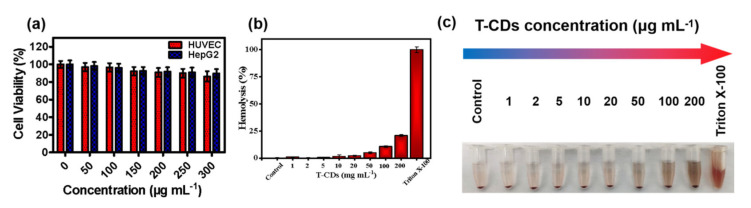
(**a**) MTT results of HUVEC and HepG2 cells viabilities after coincubation with T−CDs for 24 h at the different concentrations. (**b**) Hematological evaluation of RBCs treated with different dosages of T−CDs. The 1% Triton X−100−treated group was set as the positive control. (**c**) Photographs of RBCs treated with different dosages (0, 1, 2, 5, 10, 20, 50, 100 or 200 μg mL^−1^) of T−CDs and Triton X−100.

As mentioned above, T−CDs present excellent physical and chemical properties and have great potential in biological applications, with particular attention to cancer treatment. We first analyzed T−CDs’ biosafety for practical biological applications by MTT and hemolysis experiments. As presented in Figure 5a, we selected HUVEC (human−derived normal cells) and HepG2 (human−derived cancer cells) as models to evaluate T−CDs’ cytotoxicity. The MTT results suggested that different concentrations of T−CDs co−cultured with normal cells and cancer cells for 24 h did not show significant cytotoxicity. The cell viability was still higher than 85% even at a high concentration of 300 μg mL^−1^, proving that T−CDs have no significant cytotoxicity in the concentration range of 0–300 μg mL^−1^, which is suitable for further cell experiments.

Subsequently, fresh mouse blood was taken to evaluate the hematological toxicity of T−CDs. Figure 5b shows that in the range of 0–50 μg mL^−^^1^, the hemolysis rate of T−CDs is less than 5%. It was believed that the absorbance of the sample at a high concentration, the osmosis effect caused by this concentration, and some minor red blood cell damage contributed to the increase in hemolysis rate. In that case, the red blood cells rupture the release of hemoglobin, leading to the sample solution turning red. At the same time, Figure 5c shows that the color of the T−CDs solution at all concentrations did not turn significantly red compared with the positive control group, demonstrating that T−CDs have no considerable hematological toxicity. Red blood cells are relatively intact in T−CDs solution.

### 2.5. Photodynamic Application of the T−CDs

**Figure 6:**
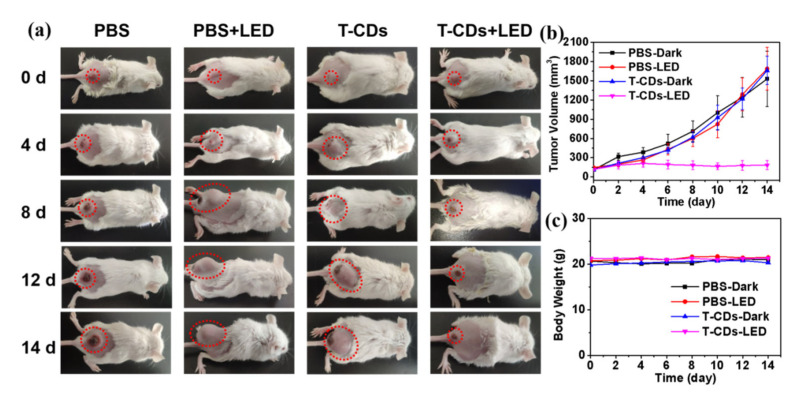
(**a**) Photographs of 4T1−tumor−bearing mice on different days post various treatments. (**b**) Tumor volume changes in the indicated groups during treatment. (**c**) Body weight changes in the indicated groups during treatment.

We used 4T1 cells to subcutaneously create a typical in vivo tumor model in the mouse buttocks and back in order to examine the in vivo photodynamic treatment effect of T−CDs. As illustrated in Figure 6a, tumor volume increased significantly over time in either the phosphate buffered saline (PBS), PBS + LED or T−CDs groups, while only the T−CDs + LED significantly inhibited tumor growth. This result is also more clearly demonstrated by the quantitative data plot of tumor volume in Figure 6b, indicating that T−CDs exhibited significant antitumor activity against solid mouse tumors under LED light irradiation, which is consistent with the results of the EPR and in vitro PDT experiments described above. Furthermore, it can be seen from the body weight curve of the mice that the weight of mice in the T−CDs + LED group has no distinct fluctuation compared with the other three groups, which means that the treatment with T−CDs has no prominent effect on the body weight and growth of the mice (Figure 6c).

The tumor cells were extracted from mice after 14 days of therapy. T−CDs were further investigated for their biocompatibility as well as their potential to cause cellular and pathological changes in major organs as a result of PDT treatment (heart, liver, spleen, lungs, and kidney). Histopathological analysis was performed using hematoxylin−eosin (H&E) staining [2,35]. As shown in Figure S4, compared with several other control groups, the normal organ tissues hardly showed any pathological changes after PDT treatment of mouse tumors with T−CDs. Only in the tumor area, after the tumor was treated with T−CDs under LED, pathological cellular changes appeared, and PDT resulted in more areas of tumor necrosis. This study confirmed the MTT results, which further demonstrated that T−CDs are robust and secure carbon nano−photosensitizers because of their low biotoxicity, good biocompatibility, ability to generate ·OH effectively, and outstanding anti−tumor properties in vivo and in vitro. 

### 2.6. Biological Imaging Application of the T−CDs

**Figure 7:**
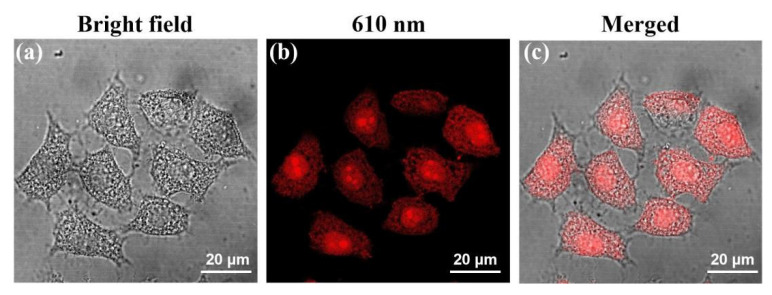
CLSM images of HepG2 incubated with 200 μg mL^−1^ T−CDs for 2h. The images were obtained under (**a**) bright−field, (**b**) 610 nm excitation and (**c**) merge from left to right (Scale bar = 20 μm).

Due to T−CDs good biocompatibility, low cytotoxicity, and bright fluorescence emission in the red region, we studied the application of T−CDs in the field of bioimaging. First, HepG2 cells were selected as the model for cell imaging. T−CDs (200 μg mL^−1^) were co−incubated with HepG2 cells for 2 h, and the cell uptake capability were observed by laser confocal microscopy. Figure 7 shows the bright field of T−CDs and HepG2 cells after co−culture, the fluorescence field under 610 nm excitation, and the combined photos of the two cells. The cell morphology is good under the bright field. When stimulated by excitation light at 610 nm, the whole cell showed bright red fluorescence, T−CDs; even entered the nucleus, enriched in the nucleolar region, and showed brighter red fluorescence emission. According to the previous reports in literature [32,36,37,38], the rich surface group of CDs allows them to efficiently pass through the cell membrane and stain cytoplasm, then enter the nucleus, and finally localize in the nucleolar region. This phenomenon may be related to ribosomes RNA in the cytoplasm and nucleus of tumor cells. The above laser confocal results showed that T−CDs could stain the cytoplasm and even enter the nucleus to enrich and stain the nucleoli within 2 h, which has a potential usage as a red fluorescent cell imaging probe.

**Figure 8:**
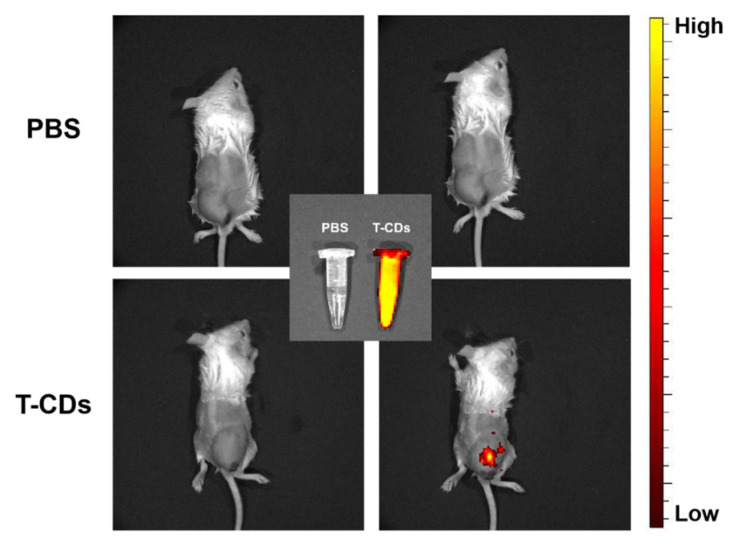
In vivo fluorescence images of mice treated with intratumoral injection of PBS and T−CDs (λ_ex_ = 600 nm, λ_em_ = 670 nm). (Inset: fluorescence images of PBS and T−CDs solution.).

In order to study the imaging effect of T−CDs in vivo, we selected tumor−bearing mice cultured with 4T1 cells as tumor models in vitro. 100 μL PBS solution and 200 μg mL^−1^ T−CDs were injected into the tumor subcutaneously. As shown in Figure 8, the images collected by the small animal live fluorescence imager showed higher intensity fluorescence emission at the mouse tumor site at 600 nm after injection of T−CDs compared with PBS, indicating that T−CDs could penetrate the epidermal tissue and have excellent development effects at the solid tumor site in vivo. Meanwhile, the pure solution of T−CDs also showed a strong signal under the in vivo imager, while the PBS solution showed no signal strength (inset in Figure 8).

These results indicate that T−CDs have a good biological imaging effect in the red light region and can cross the nuclear membrane and accumulate in the nucleolus region. Meanwhile, in vivo imaging studies have proved that the red fluorescence of T−CDs can achieve excellent imaging effect at the subcutaneous tumor site after intratumoral injection.

## 3. Materials and Methods

### 3.1. Materials

All chemicals used in the experimental procedure were purchased directly from the market and were not purified further. The citric acid (CA), 5,5−dimethyl−1−pyrroline N−oxide (DMPO), 4−Amino−2,2,6,6−tetramethylpiperidine (TEMP) and 3−(4,5−dimethylthiazol−2−yl)−2,5−diphenyltetrazolium bromide (MTT) were purchased from Aladdin Industrial Corporation (Shanghai, China). Formamide, methanol, acetone, and dimethyl sulfoxide (DMSO) were purchased from Sinopharm Group Chemical Reagent Co. Tea polyphenols were purchased from Zhongshan Huazhong Food Additives Co., Ltd. (Zhongshan, China).

### 3.2. Synthesis of T−CDs

It is synthesized from citric acid (CA) and tea polyphenols by a solvothermal reaction in the reaction system with formamide as a solvent. First, weigh 1.5 g anhydrous CA and 1 gtea polyphenol powder, add 30 mL formamide solvent, and sonicate for 10 min to thoroughly mix the reaction materials. Subsequently, it was transferred to a 50 mL autoclave, heated at 180 °C in a muffle furnace for 4 h, and the heating rate was set to 2 °C min^−1^; the cooling rate was 5 °C min^−1^. After the reaction has cooled, remove the dark brown T−CDs stock solution. Add 100 mL acetone to 30 mL of the above T−CDs reaction stock solution and sonicate for 20 min to make it thoroughly mixed (the addition of acetone will cause precipitation), and cool it in a −20 °C refrigerator overnight. The above solution precipitation was collected by suction filtration, and the precipitation was successively washed with acetone solutions containing 10%, 20%, and 30% methanol. The precipitates were dissolved repeatedly in a pure methanol solution, and the clear solution was collected until the solution was colorless. Finally, the collected methanol solution of T−CDs was rotary evaporated at a temperature of 45 °C to obtain a purified black T−CDs powder sample.

### 3.3. Characterization

T−CDs morphology was characterized by TEM (JEM−2100), and the crystallinity and disorder of T−CDs samples were analyzed by confocal laser Raman spectroscopy (LabRAM HR800). Both X−ray photoelectron spectroscopy (XPS, ESCALAB250 spectrophotometer) and Fourier transform infrared spectroscopy (FTIR), utilizing a Nexus 870 FTIR (Thermo Nicolet, Waltham, MA, USA) spectrometer, were employed to assess the structure and composition of the samples. The capture of free radical signals using Electron paramagnetic resonance (EMXnano) spectroscopy. The average molecular weight (Mw, Mn) and polydispersion index (PDI) of T−CDs were determined by the GPC (Waters 2414, Milford, MA, USA) method with 0.1 mol L^−1^ sodium nitrate solution as eluate at a flow rate of 1 mL min^−1^. Then the GPC curve was analyzed by the Breeze software. UV−Vis absorption and PL spectra were recorded using a UV−1800PC spectro−photometer and a fluorescence spectrophotometer (F−47000, Hitachi). Fluorescence images were recorded using a confocal laser scanning microscope (CLSM) (Leica TCS SP8X). A classical MTT assay measures cytotoxicity by absorbance zymography (Molecular Devices, CMAX PLUS, SpectraMax^®^ Absorbance Reader, Shanghai, China).

### 3.4. Detection of the Reactive Oxygen Species (ROS)

Analysis of reactive oxygen species and their ability to generate T−CDs under light stimulation by EPR spectroscopy, using DMPO as the trapping agent for OH; TEMP as the ^1^O_2_ capture agent. Using pure water as a control, the ability of T−CDs aqueous solution to generate OH and ^1^O_2_ before and after irradiation was tested. A white light LED (wavelength 400–500 nm, 15 mW cm^−2^) was used as the excitation light source, and the illumination time was 12 min.

### 3.5. Cell Culture and Cell Experiment

In the in vitro experiment, 4T1 cells, HUVEC cells and HepG2 cells were used as cell models. The cell culture medium was high−glucose DMEM medium (containing 1% penicillin/streptomycin and 10% fetal bovine serum), cultured in humid air containing 5% CO_2_ in an incubator at 37 °C. The cells were digested and passed through trypsin to keep the cells in good condition during the cell experiment.

#### 3.5.1. Cytotoxicity Test

Using MTT colorimetry, the impact of T−CDs on cell viability was evaluated. Aspirate the growth media and add it to each well after the cells have been incubated in a 96−well culture plate for around 12 h in a 5% CO_2_ atmosphere (0.5~1 × 10^4^ cells, 100 μL per well). The T−CDs solutions were incubated for 24 h at various concentrations (0, 50, 100, 150, 200, 250, and 300 g mL^−1^, diluted with the prepared medium). Then, after incubating each well with 10 μL of MTT solution (5 mg mL^−1^) in the dark for 4 h, aspirate the solution from each well, add 150 μL of DMSO, and shake the mixture at room temperature for 5 to 10 min. Finally, use 563 nm as the detection wavelength and 630 nm as the reference wavelength to measure the absorbance in a microplate reader. 

4T1 cells were chosen as the model for the in vitro PDT experiment following the MTT experimental methods mentioned above; however, 12 h after adding the T−CDs sample co−culture, an extra step was taken to pass a white light LED (wavelength 400–500 nm, 15 mW cm^–2^) as the light source. Continue to culture the cells for another 12 h after exposing the 96−well plate to radiation for 12 min. A 96−well plate was used for each study, containing six replicate wells for each sample concentration.

#### 3.5.2. Biological Imaging Stained with the T−CDs

HepG2 cells, selected as the cellular imaging model, were plated on 15  mm laser confocal dishes with an initial density of 6 × 10^4^ cells per mL and allowed to adhere for 12  h. Then, the culture medium was replaced by T−CDs (200  μg·mL^−1^) for another 2  h incubation at 37  °C. The cellular fluorescence images were acquired after washing with PBS for 3 times on the laser scanning confocal microscope.

#### 3.5.3. Hemolysis Test

Take 500 μL of fresh mouse blood (with a small amount of anticoagulant) and centrifuge at 2000 rpm for 5 min to remove the supernatant; resuspend the bottom red blood cells with 1 mL of normal saline, continue to centrifuge at 2000 rpm for 5 min to remove the supernatant, repeat 3–4 times until the supernatant is almost colorless, and finally, resuspend the red blood cells in 1 mL of normal saline. Take 50 μL of the above red blood cell suspension, and add T−CDs to the final concentration of 1, 2, 5, 10, 20, 50, 100, and 200 μg mL^−^^1^, the final volume of the solution is 400 μL, the negative control is red blood cells resuspended in In normal saline, the positive control is red blood cells resuspended in 0.5% (wt) Triton X−100; the above 10 centrifuge tubes were incubated at 37 °C for 2 h, centrifuged at 2000 rpm for 5 min to take pictures, and 100 μL of the supernatant was taken in a 96−well plate to measure the absorbance at 450 nm using a microplate reader. Calculated by the following formula:

(1) $$\mathrm{Hemolysis}\;\mathrm{rate}\;\%\;=\frac{A_{0}-A_{2}}{A_{1}-A_{2}}\times 100\%,$$

(A_0_: Sample absorbance; A_1_: Positive control absorbance; A_2_: Negative control absorbance)

### 3.6. Animal Culture and Animal Experiments

Select female Balb/c mice (specific pathogen−free animals, SPF grade), aged 4–6 weeks, weighing about 20 g as the model for animal experiments. Using 4T1 cells as cancer cells in a mouse solid tumor model, inject 100 μL of a cell suspension containing about 2–10 × 10^7^ 4T1 cells into the buttocks and back of each mouse. The mice were cultured for about 6–7 days under 12 h light and 12 h dark per day after injection, and tumor−bearing mice were used for in vivo imaging and in vivo PDT experiments after solid tumor growth and formation.

In vivo PDT experiment: 12 mice with relatively average tumor size were randomly divided into 4 groups. The in vivo PDT effect of T−CDs and control sample PBS was investigated. First, 100 μL of 200 μg mL^−1^ of T−CDs solution was injected into the tumor area of 1 group of mice. Then, the tumor area was irradiated with LED (400–500 nm, 15 mW cm^−2^) for 12 min, and the control group was T−CDs were injected without light, PBS with light for 12 min, and PBS without light. Treatment was performed every two days, and changes in body weight and tumor volume of mice were recorded to evaluate the effect of treatment and the effect of treatment on the body weight of mice. Calculate the tumor size as follows:

(2) $$V=\frac{1}{2}\;\times \;(L\;\times \;W^{2})$$

(V: tumor volume, L: tumor length, W: tumor width).

After 14 days of treatment, the mice were sacrificed to obtain their organs (heart, liver, spleen, lung, kidney, and tumor) and soaked in 4% paraformaldehyde for preservation. Embed in paraffin, section thickness 4–6 μm. Sections were stained with hematoxylin and eosin (H&E) for histopathological evaluation to further assess T−CDs’ safety.

## 4. Conclusions

In summary, the solvothermal method was successfully applied to prepared T−CDs with red and blue dual emissions by tea polyphenols compounds as the precursor. The surface of the T−CDs was rich in polyphenol groups through the introduction of tea polyphenols, and it had good dispersion in water and a biological medium. Additionally, hemolytic and MTT experiments showed that T−CDs exhibited high biocompatibility in vitro and minimal cytotoxicity (regular and cancer cell lines). EPR spectral analysis showed that T−CDs could efficiently generate ·OH under LED light stimulation. While using the 4T1 cells as the model, it can be seen that T−CDs could effectively inhibit the proliferation of cancer cells in both cell and solid tumor models, showing an excellent PDT effect. What is more, T−CDs had no obvious effect on the main organs of the mice and did not produce any lesions. Therefore, the T−CDs can be used as a type I PS for the PDT of cancer cells.

## Data Availability

The data presented in this study are available on request from the corresponding author.

## Supplementary Materials

The following supporting information can be downloaded at: https://www.mdpi.com/article/10.3390/molecules27238627/s1, Figure S1: Molecular weight distribution of T−CDs determined by GPC; Figure S2: Absolute fluorescence quantum yield of in H_2_O under (**a**) 360 nm and (**b**) 610 nm excitation; Figure S3: Corresponding quantitative curve of cell viability for MTT results of the 4T1 cells treated with T−CDs in the dark and under LED light irradiation (12 min); Figure S4: Histological analysis of heart, liver, spleen, lung, kidney, and tumor, of the mice for different groups after PDT (scale bar = 50 μm).

## Sample Availability

Samples of the compounds are not available from the authors.
