# Supplementary material for: Carbon Dots Derived from Tea Polyphenols as Photosensitizers for Photodynamic Therapy
Source: Molecules. 2022 Dec 6;27(23):8627. doi: 10.3390/molecules27238627 (PMC9736769; doi:10.3390/molecules27238627)
Supplement: Supplementary file 1 [file molecules-27-08627-s001.zip › molecules-2042993-supplementary.pdf]

Supporting Information

Carbon dots derived from tea polyphenols as photosensitizers for photodynamic therapy

Yuxiang Yang [1], Haizhen Ding [1], Zijian Li [2], Antonio Claudio Tedesco [1,3] and Hong Bi [2,*]

[1]School of Chemistry and Chemical Engineering, Anhui University, 111 Jiulong Road, Hefei 230601, China;

[2]School of Materials Science and Engineering, Anhui University, 111 Jiulong Road, Hefei 230601, China;

[3]Department of Chemistry, Center of Nanotechnology and Tissue Engineering−Photobiology and Photomedicine Research Group, Faculty of Philosophy, Sciences and Letters of Ribeirão Preto, University of São Paulo, São Paulo 14040−901, Brazil

*Correspondence: bihong@ahu.edu.cn

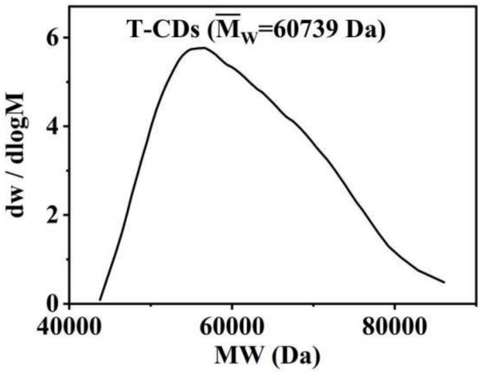

Figure. S1 Molecular weight distribution of T-CDs determined by GPC.

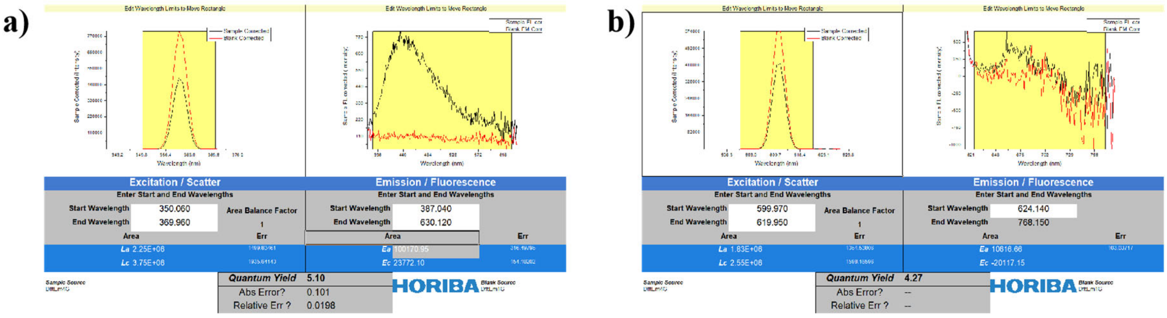

Figure. S2 Absolute fluorescence quantum yield of in H_2O under a) 360 nm and b) 610 nm excitation

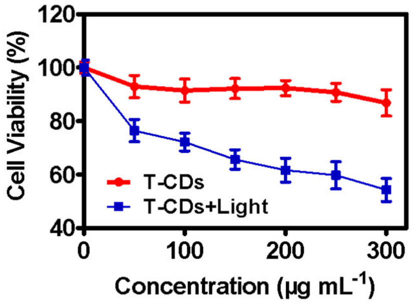

Figure. S3 Corresponding quantitative curve of cell viability for MTT results of the 4T1 cells treated with T-CDs in the dark and under LED light irradiation (12 min).

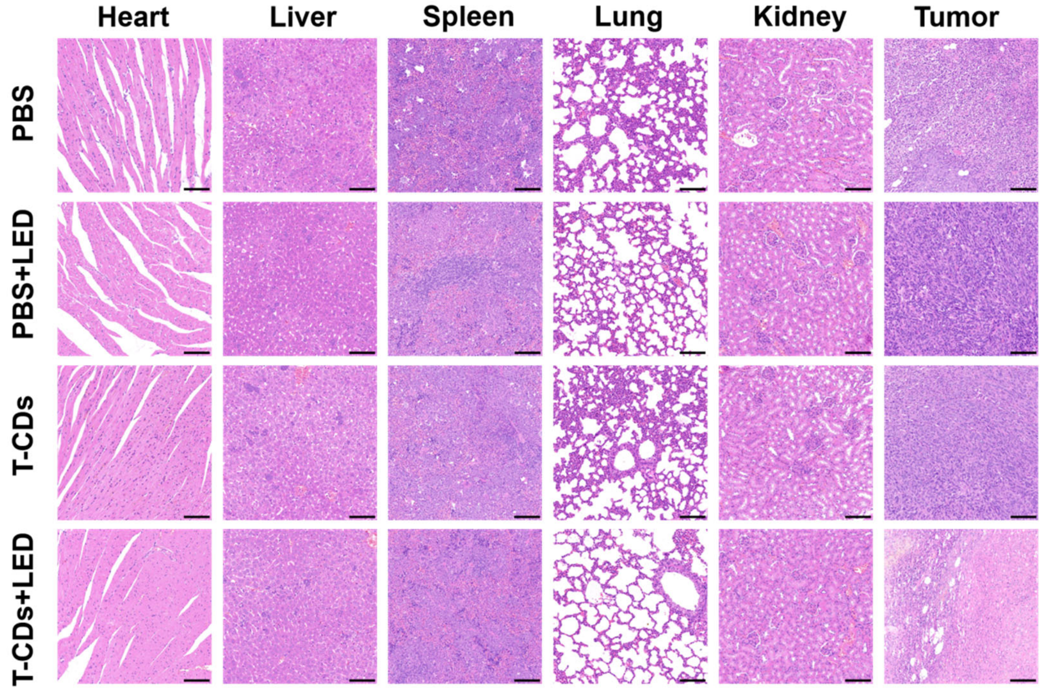

Figure. S4 Histological analysis of heart, liver, spleen, lung, kidney, and tumor of the mice for different groups after PDT (Scale bar = 50 μm)
